# Interest-parity conditions during the era of the classical gold standard (1880–1914)—evidence from the investment demand for bills of exchange in Europe

**DOI:** 10.1186/s41937-017-0008-5

**Published:** 2018-05-01

**Authors:** Nils Herger

**Affiliations:** Study Center Gerzensee, Dorfstrasse 2, P.O. Box 21, Gerzensee, 3115 Switzerland

**Keywords:** Bills of exchange, Exchange rates, Gold standard, Interest-parity condition, F31, N13, N23

## Abstract

This paper examines interest-parity conditions that arguably held as regards the investment demand for bills of exchange during the classical gold standard (1880–1914). Contemporaneous guides to the foreign exchanges report that close connections between the exchange and discount rates arose mainly with bills traded in London and the major financial centres on the European continent. As implied by the interest-parity condition, and in particular when future exchange-rate movements were covered by a suitable long-bill transaction, weekly data do suggest that between Paris, Amsterdam, Berlin, Brussels, and London, the return from discounting bills of exchange in the local money market was roughly equivalent to the return from investing in foreign currency bills.

## Introduction

“The Foreign Exchanges [...] are the barometer of the Money Market. Between the price of London bills, as expressed in the current rate of discount, and the price of foreign bills, as expressed in the current exchange rate, there exists at times a close sympathy [...].” Clare (1902, p. 87)

The introductory quote is not remarkable for emphasising the close connection between the foreign exchange and money markets, which features prominently in the interest-parity condition, but for having been taken from a guide first published back in 1891 to provide “A Money-Market Primer and Key to the Exchanges”. The author, George Clare, wrote this book for the practitioner in the banking industry. Although Clare never reached academic fame, his work was praised by none other than Alfred Marshall as “the only tolerably good small book dealing with Banking and The Exchanges” (Groenewegen 1985). Apparently, before the year 1900, bankers had already developed a solid understanding of the connections between the exchange and interest rate (or at the time the discount rate). Indeed, according to Einzig (1962, pp. 184–185), during the nineteenth century, “the establishment of closer relations between banks [...] led to a considerable increase in the volume of exchange arbitrage [...] and also the volume of interest arbitrage” and the corresponding techniques were “described in detail in innumerable practical books and articles”. Specifically, it was arguably mainly in the economically and financially most advanced European nation states such as Britain, France, Germany, Belgium, and the Netherlands, where banks had the option of reinvesting idle deposits in the local money market, or earning short-term interest by purchasing bills of exchange involving foreign currencies. Reflecting the importance of this so-called investment demand for bills of exchange, numerous contemporaneous books can be found discussing interest-parity transactions involving European bills of exchange (Clare 1895, ch. 14–16, 1902, ch. 9; Spalding 1915, ch. 7; Thomas 1921, ch. 8). In his famous portrait of the London money market in Lombard Street, even Bagehot (1873, pp. 45–46) touches briefly on this issue.

By way of contrast, around 1900, academic research about the foreign exchanges was still in its infancy. Early fragments of the interest-parity condition can already be found in Thornton (1802, ch. 5), whereas Goschen (1861) and Lotz (1889) published more extensive, but still highly informal, discussions. It was only during the first part of the twentieth century when formal theories that exchange rate movements offset, on average, the differences in interest rates between currencies began to establish themselves in the academic literature (Einzig 1962, ch. 17 and 21). In particular, Fisher (1896, ch. 9) and Keynes (1923, ch. 3.4) are often quoted as path-breaking contributions relating to, respectively, the uncovered and covered interest-parity condition.

Compared with the voluminous empirical literature testing the interest-parity condition with modern data, for the major European financial centres during the era of the classical gold standard, the corresponding question has hitherto received much less attention. Although a small empirical literature including Goodhart (1969) and Coleman (2012) has tested the uncovered interest-parity condition around the year 1900, these contributions have looked at the dollar-to-sterling exchange rate and, owing to the slow dissemination of information and substantial transaction costs, found only scant support for the interest-parity condition. However, at the end of the nineteenth and the beginning of the twentieth century, interest arbitrage arose arguably primarily as regards the investment demand for bills of exchange issued in London and in the main financial centres across the English Channel. Corroborating this, the contemporaneous guides emphasise that, rather than with New York, close links existed between the discount rate in the open money markets of Paris, Berlin, Amsterdam, and Brussels and the demand for London bills of exchange (Clare 1902, pp. 129ff.). As regards the covered interest-parity condition, Flandreau and Komlos (2006) provide an empirical study about early arbitrage transactions between Berlin and Vienna, where a forward foreign exchange market had already developed during the second half of the nineteenth century. Conversely, for the major currencies around the world, regular forward exchange transactions did not occur until after the First World War (Einzig 1962, pp. 182–183, 1967, pp. 7–8). Finally, rather than testing interest-parity relationships, they have also been employed to extract implied, or shadow, interest rates from international bills of exchange transactions. This approach, which takes the interest-parity condition for granted, has mainly been used for the pre-modern era, for which interest rate data are often unavailable and, when they were reported, were often subject to the constrains from usury laws (see e.g. Flandreau et al., 2009). However, Obstfeld and Taylor (2004, pp. 87ff.), for the case of London, New York and Berlin, and Flandreau and Rivière (1999), for the case of London and Paris, present corresponding results for the late nineteenth century.

Against this background, this paper endeavours to contribute to the literature by testing how closely the discount and exchange rates were connected between the leading European financial centres during the era of the classical gold standard (1880—1914). In so doing, the fact that bills of exchange were the dominant security for short-term investments and managing exchange-rate risk will be taken into account. Based on weekly data, the results suggest that the bills of exchange versions of the interest-parity condition held remarkably well between European currencies, which were known for their long-standing stability as regards the convertibility into gold. Specifically, when comparing the continental discount rate with the exchange-rate adjusted return in London, the postulated proportional relationship tends to arise. Furthermore, even for investments by a London-based bank on the continent, where the lack of suitable long-bills made it difficult to hedge against future exchange-rate movements, the interest-parity condition holds reasonably well.

The paper is organised as follows. The next section explains the historical role of bills of exchange for determining the relevant market exchange and discount rates during the gold standard. The “Weekly discount and exchange rate data” section discusses the data. The “Methods” section explains the peculiarities of interest-parity conditions involving bills of exchange. The “Results and discussion” section introduces the econometric strategy and presents the results. The “Conclusion” section summarises and concludes.

## Background

### Exchange rates during the gold standard

From around 1880 until the outbreak of the First World War in 1914, the gold standard served as role model for the international currency system. During this era of the classical gold standard, the definition of the currency value in terms of gold—the so-called mint-par—gave rise to officially fixed exchange rates. Nevertheless, the rates on the foreign exchange markets did move to some degree. One reason was that international gold shipments, which enforced the mint-par, were costly, wherefore the market exchange rate could fluctuate within a band of gold-arbitrage inactivity (see e.g. Clare 1895, ch. 7). An ongoing debate is still trying to determine the width of this band, which was delimited by the so-called gold-points. There are several reasons why this has turned out to be challenging including the numerous, time-varying cost components to transport gold across countries (Clare 1902, pp. 78–79; Canjels et al. 2004, p. 869), or the complications arising when triangular gold arbitrage is possible (Coleman 2007).^1^ To nevertheless provide some rough values, for the French and the Belgian franc, contemporaneous sources set the upper and lower gold-points at, respectively, 25.12 Fcs/£ and 25.32 Fcs/£ (Clare 1895, p. 126; Tate 1908, p. 51), for the Dutch guilder at 12.05 Fl/£ and 12.15 Fl/£ (Tate, 1908, p. 328), and for the German mark at 20.32 M/£ and 20.53 M./£ (Clare 1895, p. 131) reflecting deviations of between 0.2 and 0.7 per cent from the mint-pars of, respectively, 25.22 Fcs/£, 12.07 Fl/£, and 20.43 M/£. Furthermore, even in its heyday, the gold standard was not a homogeneous system. In Europe, Britain and Germany came closest to the theoretical ideal of a freely convertible monometallic currency backed by gold, followed by Belgium, France, the Netherlands, and Switzerland, where the conversion of the local currency into gold was subject to some restrictions (see Eichengreen 2008, pp. 20ff.). Still, for decades, these relatively wealthy and financially advanced European nation states managed to anchor the exchange rate to the mint-par, which arguably implied that the remaining, small fluctuations on the foreign exchange market were primarily a reaction to international differences in interest (discount) rates (Clare 1902, p. 94). Conversely, the currency systems of other European countries were at most incompletely associated with the gold standard. For example, aside from a short period in the 1880s, the Italian mint-par of 25.22 lire/£ was never officially instituted (Eichengreen 2008, p. 17). In Spain, Portugal, or Russia, substantial parts of the currency consisted of inconvertible paper money (Clare 1895, pp. 157–160). Austria-Hungary had essentially a freely floating currency until the end of the 1880s, and undertook several monetary reforms during the 1890s to gradually stabilise the exchange rate against gold around the year 1900 (see Von Mises 1909; Flandreau and Komlos 2006). The exchange rates of all these countries were relatively instable (Einzig 1962, 198–199) and reacted to fluctuations in international trade, or changes in foreign indebtedness (Clare 1902, p. 94).

### Bills of exchange are the key financial instrument

Amid an era of widespread economic and political stability, the second half of the nineteenth century witnessed an unprecedented expansion of cross-border trade and capital flows (Obstfeld and Taylor 2004). However, owing to the costs as well as the inelastic supply of gold-backed money, the massive increase in international payments was not matched by a corresponding increase of cross-border transfers of gold bullion or coins. Hence, international capital flows exceeded by far the volume of trade, which was, in turn, far larger than cross-border transfers of gold (Eichengreen 2008, pp. 24ff.). These discrepancies reflect that, since the Middle Ages, bills of exchange were widely used to finance international payments (see, e.g. Denzel 2010, ch. 3, Einzig 1962, ch. 7). In essence, a bill of exchange was a written order by an issuer, called the drawer, instructing a counterparty^2^, called the drawee, to pay a certain amount of money at a specific place either immediately (sight-bill) or at the end of a given—usually 3 months—term to maturity (long-bill).

Bills could be issued requiring payment in a foreign country or city with a different currency. Since they dominated international payments during the nineteenth century, bills of exchange determined the relevant foreign currency price for cross-border business (see, e.g. Denzel 2010, ch. 3.3). As such, the market exchange rates quoted for foreign bills could deviate from the mint-par. More specifically, when the demand for bills on a foreign currency was relatively high and/or the supply relatively low, the market exchange rate of that currency appreciated. What will be important for interest parity considerations is that fluctuations of the market exchange rate occurred with sight as well as with long-bills.

By combining elements of credit with money transfers, bills of exchange became the preferred instrument to arrange cashless payments. Though originally designed to finance trade (trade-bill), around 1900, bills of exchange served also as instrument for purely financial purposes such as making short-term foreign investments (finance-bill) (Goschen 1861, ch. 3; Escher 1913, ch. 2; Clare 1895, ch. 13). In particular, banks on the European continent used to reinvest substantial parts of the savings deposited with them by purchasing bills issued in London rather than in their local money market. This practice became known as “the continental investment demand for London bills” (see Clare 1902, ch. 9; Spalding 1915, ch. 7; Thomas 1921, ch. 8). Conversely, before the First World War, British banks tended to ignore foreign currency bills (Clare 1895, p. 89, 1902, p. 95; Thomas 1921, p. 78).

Especially when bills of exchange were drawn on a bank with a good reputation, they were seen as safe asset, or “first-class” bank-paper. This provided the basis for turning a bill into a transferable financial instrument, which could be sold well before its due date to a third party, often a discount house, which purchased a bill at a discount in anticipation of receiving the final payment (see Cassis 2010, p. 84). From the perspective of the drawer, the selling (or discounting) of bills had the advantage of receiving early payment, but came at the price of the so-called discount rate, that is the interest charged by the third party. The development of discount markets and specialised discount houses meant that bills of exchange became tradable and, hence, an “admirably liquid security” (Spalding 1915, p. 80). Due to the pivotal role of bills of exchange, the discount rate used to be the most closely watched interest rate of the financial system.

### London serves as global discount market

Reflecting the role of Britain as leading industrial nation, during the four decades preceding the First World War, London had established itself as principal hub for arranging, funding, and insuring the bulk of international trade and payments (Cassis 2010, pp. 83ff.). Though other financial centres—in particular, Paris and Berlin in Europe and New York in America—also witnessed rapid developments, the dominance of London was such that, prior to 1914, around half of global trade was financed by bills denominated in sterling, which had obtained the status of international currency par excellence (Atkin 2005, p. 5). With bills of exchange accounting for most international payments, this implied that a group of British merchant banks and discount houses accepted and discounted vast amounts of sterling-bills (generally of three months maturity) sustaining the most liquid money market in the world (see e.g. Fletcher 1976, ch. 2). Banks of the European continent, through their London branches or agents, took part in this market (Thomas 1921, pp. 80ff.), and thanks to the relatively unhindered flow of capital and the technological progress in telecommunication (telegraph, telephone), financial centres became closer intertwined and a genuine international capital market began to emerge (Cassis 2010, p. 131).

**Fig. 1:**
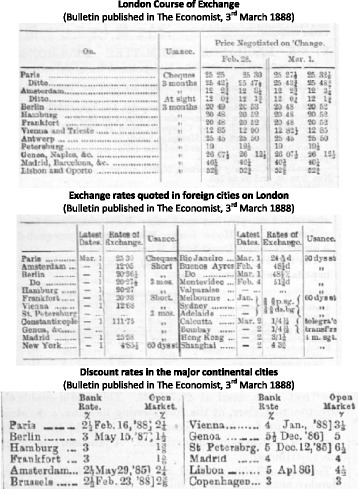
Exchange and interest rates on 1 March 1888

Against this background, the interest and exchange rates set in London served as an international landmark. The corresponding data were published, typically on a weekly basis, in the financial press in Britain and abroad. Figure 1 provides examples taken from the 3 March 1888 edition of The Economist. The top panel shows the London Course of Exchange bulletin, which reports the exchange rates on various foreign cities for the two most recent trading days (here 28 February and 1 March 1888) at the Royal Exchange, which was the main market for foreign currency bills in Britain (see Clare 1895, ch. 8). For each foreign city, two quotations are given. The first (better) rate refers to “first-class paper”, which generally meant bills of exchange involving banks with a good reputation, whereas the second (higher) rate applied to ordinary trade bills involving little-known firms (see, e.g. Clare 1895, p. 41; Spalding 1915, pp. 50–51). When contemplating the actual exchange rate data in the “Weekly discount and exchange rate data” section, it will be important to remember that banks preferred first-class bills (Thomas 1921, ch. 8; Clare 1895, p. 90; 1902, p. 98f.). Of note, most exchange rates in London refer to 3-month bills; sight or cheque rates were often only quoted on Paris and sometimes Amsterdam (Clare 1902, pp. 82, 85). Conversely, according to the middle panel, sight exchange rates quoted abroad for bills payable in London existed for several European financial centres including Paris, Amsterdam, as well as Berlin (and sometimes Brussels and Vienna). Finally, the London and continental open money market discount rates were also published in The Economist (see bottom panel of Fig. 1).

## Weekly discount and exchange rate data

From tables such as those of Fig. 1, for the decades before the First World War, Neal and Weidenmier (2003) have collected weekly time series of discount and exchange rates across a range of financial centres. Recall that, whereas most London exchange rates referred to long-bills, many financial markets on the European continent quoted only sight rates on London. More specifically, for the 1880 to 1914 period (the common sample ends in December 1913), the data include money market discount rates for French francs in Paris, for Belgian francs in Brussels, for Dutch guilders in Amsterdam, and for German marks in Berlin (denoted here by $i_{t}^{*}$) as well as for sterling in London (denoted here by *i*_*t*_). Furthermore, there are exchange rates derived from sight-bills issued in a continental financial centre (and currency) and payable in sterling in London within a week ($S_{t}^{*}$). For example, for Paris and London, this exchange rate was briefly referred to as the “sight-rate in Paris on London”. Furthermore, there are exchange rates derived from long-bills issued in London and payable in a specific continental city (and currency) in 3-month time (*L*_*t*_). For example, for London and Paris, this exchange rate was briefly referred to as “long-rate in London on Paris”. Detailed definitions and sources of the data can be found in Table 1.

**Table 1:** Description of the data set

Variable	Unit	Description
$S_{t}^{*}$	Continental currency per £ (in logarithms)	Exchange rate in terms of the price of a sight-bill payable in sterling as traded in continental currency. In brief, these bills were called “sight-bills on London” and the corresponding exchange rate was called the “sight rate in a given continental city on London”. The data are available for sight-rates in Paris, Amsterdam, Berlin, and Brussels. For the case of Paris and Brussels, the cheque rate has been used for $S_{t}^{*}$. For the case of Brussels, the data are only partly available before 1902.
*L*_*t*_	Continental currency per £ (in logarithms)	Exchange rate inherent in a long-bill of exchange payable in continental currency as traded in sterling. The term to maturity is three months. In brief, these bills were called “long-bills in London” and the corresponding exchange rate was called the “long-rate in London on a given continental city”. For the case of Belgium, the long-rate was reported on Antwerp instead of Brussels.
*i*_*t*_	Per cent (annualised)	Interest on a short-term investment in the London money market. The interest arises from discounting a sterling bill of exchange in the open money market.
$ i_{t}^{*}$	Per cent (annualised)	Interest on a short-term investment in the continental money market. The interest arises from discounting a bill of exchange denominated in local currency in the local open money market.

Variables refer to transactions between London and four continental financial centres (Paris, Amsterdam, Berlin, Brussels) between 1880 and 1914. The data have a weekly frequency Source of the data: Neal-Weidenmier Gold Standard Database, Neal and Weidenmier (2003)

**Fig. 2:**
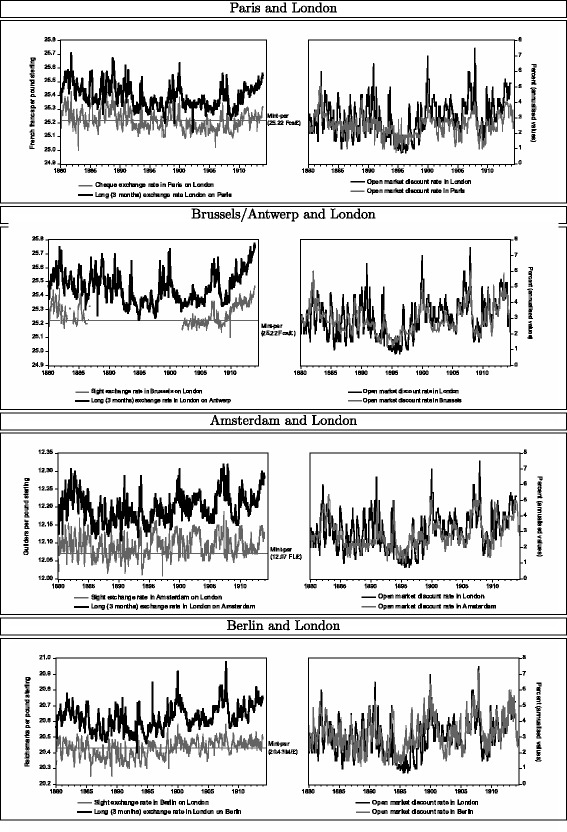
Discount and exchange rates

Contemplating first the case of Paris, which was the most important financial centre after London (Cassis 2010, pp. 101ff.), the top left panel of Fig. 2 depicts the exchange rates derived from sight-bills in Paris on London as well as from long-bills in London on Paris. Since banks invested preferably in first-class paper, these rates refer to the lower value reported in Fig. 1. As mentioned above, the sight (or cheque) rates fluctuated around the mint-par of 25.22 Fcs./£. Furthermore, since the long-rate of exchange is always above the sight-rate, a buyer of a long-bill in London could be almost certain to earn an implicit return in terms of receiving more French francs than he would have to lay out for a sight-bill. The top right panel of Fig. 2 depicts the interest rates derived from discounting bills in the open money markets of London or Paris. Although these rates follow each other quite closely, the London rate was more volatile.

Aside from France, also Belgium, the Netherlands, and Germany were reportedly important participants in the investment demand for London bills of exchange (see, e.g. Clare 1895, p. 90). Being based on a very similar international currency system than France, the Belgian franc also fluctuated around a mint-par of 25.22 Fcs/£ (see, e.g. Clare 1895, p. 130; Tate 1908, p. 51). However, as shown by the second panel of Fig. 2, before 1902, the sight-rate in Brussels on London was only sporadically reported in The Economist. Furthermore, the long-rate in London refers to Antwerp. Between 1880 and 1914, weekly time series with exchange rates and the discount rate are available for the Dutch guilder, whose mint-par stood at 12.07 Fl/£ throughout those years. Although well past its golden age (see Cassis 2010, p. 125), the Netherlands accounted still for a considerable investment demand for London bills (Clare 1902, p. 94). Accordingly, the corresponding panel of Fig. 2 shows the familiar behaviour of the discount and the guilder-to-sterling exchange rates. Finally, during the 1890s, Berlin became an increasingly important financial centre (Cassis 2010, pp. 108ff.; Flandreau and Jobst 2005, p. 989). The bottom panel reports the data for the German mark, whose mint-par stood at 20.43 M/£.

For the other financial centres around the world, exchange rates on sight and long-bills with respect to London were only published in a highly incomplete manner. This does not necessarily mean that these rates did not exist. Rather, the non-disclosure suggested, arguably, that for some countries certain foreign exchange transactions were “out of the ordinary course” and, hence, there was “no recognised quotation” (Clare 1902, p. 82). This seems to underscore that interest-parity transactions involving an investment demand for foreign currency bills occurred predominantly between sterling and a small number of European currencies that were at the heart of the gold standard.^3^

## Methods

Consider the standard uncovered interest-parity condition $(1+i_{t}) = (1+i^{*}_{t})\left (S_{t}/E\left [S_{t+m}^{*}\right ]\right)$. Recall that *i*_*t*_ denotes, here, the return in the London money market at the 3-month discount rate, $i_{t}^{*}$ the corresponding return in the continental money market, whereas *S*_*t*_ denotes the sight-rate in London, and $E\left [S_{t+m}^{*}\right ]$ the expected sight-rate on London upon maturity at date *t*+*m*. To eliminate uncertainties as regards the expected currency gain/loss of $S_{t}/E\left [S_{t+m}^{*}\right ]$, the covered version of interest parity relies on financial markets, on which currencies can be traded for future delivery against payment on delivery at a prearranged exchange rate. However, for most currencies, such forward markets did not develop until after the First World War (Einzig 1962, pp. 182–183, 1967, pp. 7–8)^4^. Instead, early strategies to exploit international differences in interest rates relied heavily on long-bill investments (see, e.g. Clare 1895, ch. 14–16; Spalding 1915, pp. 80ff.; Thomas 1921, pp. 78ff., Einzig 1967, p. 6). Although they give rise to an implicit forward exchange rate, it is important to recognise that long-bills differ from a modern forward contract in terms of also encapsulating a short-term credit by requiring an immediate payment from the buyer, who is promised to receive a certain amount of foreign currency back upon maturity (see Clare 1895, ch. 12). Hence, interest-parity conditions involving long-bills differ from their modern counterparts (see Juhl et al. 2006).

Contemplating the cases for which data are available, consider first the continental investment demand for bills of exchange, where a bank based, e.g. in Paris, Amsterdam, Berlin, or Brussels buys a bill on London at the sight-rate $S_{t}^{*}$ and, to hedge against exchange-rate risk, combines this with an immediate investment in a long-bill in London at the long-rate *L*_*t*_. To eliminate arbitrage opportunities, the spread $L_{t}/S_{t}^{*}$ between these rates should be equal to the return of $1+i_{t}^{*}$ in the local discount market. Jointly, this yields the interest-parity condition reflecting the continental investment demand for bills of exchange, that is

1 $$ \frac{L_{t}}{S_{t}^{*}}=\left(1+i_{t}^{*}\right).  $$

Investments in the prevalent bills could also have been combined in a different way. For example, a London bank could first buy a long-bill on a continental city. Upon maturity, the amount of foreign currency payable had to be invested in a sight-bill issued *on* London at the expected sight-rate $E\left [S_{t+m}^{*}\right ]$. For an interest parity to emerge, the resulting return had to coincide with the return of 1+*i*_*t*_ in the London discount market. Taken together, this yields what might be called the interest-parity condition reflecting the London investment demand for bills of exchange, that is

2 $$ \frac{L_{t}}{E\left[S_{t+m}^{*}\right]}=(1+i_{t}).  $$

Note that, in contrast to (1), (2) is subject to exchange-rate risk as the realised return of the long-bill investment depends on the, a priori unknown, rate of a sight-bill at future date *t* + *m*. In any case, (1) and (2) concur nicely with the historical observation that the price of a long-bill was “based upon the sight-rate, rising and falling in agreement with it [...]” (Clare 1902, p. 83) as well as that investors in long-bills had to be compensated by an implicit interest rate for awaiting payment (Goschen 1861, pp. 52ff.; Clare 1895, ch. 12; Spalding 1915, ch. 6).

## Results and discussion

This section estimates and tests the just mentioned interest-parity conditions by means of econometric methods that were unavailable around 1900. In particular, following the modern literature and applying a logarithmic transformation (with the corresponding sight and long-bill rates being denoted by lowercase letters), the regression equation approximating the continental investment demand for bills of exchange of (1) is given by

3 $$ l_{t}- s_{t}^{*} = \alpha + \beta \left(i_{t}^{*}\right)+\epsilon_{t}.  $$

Here, *α* is an intercept, *β* is a slope-coefficient reflecting how far exchange and interest rates move in tandem, and *ε*_*t*_ is a statistical error term. A scenario where there are no interest-arbitrage opportunities left in the foreign exchange and money markets implies that *β*=1. Whereas the estimation of *α* and *β* in (3) is relatively straightforward, some econometric issues arise regarding the corresponding standard deviations. In particular, a dataset combining 3-month long-bills with observations that have a weekly frequency gives rise to overlaps within the sample, which introduce moving-average terms to the residuals. To account for these when estimating coefficient standard deviations, Chinn (2006, pp. 9f.) uses a heteroscedasticity and autocorrelation (HAC) robust variance-covariance matrix towards a fixed length of serial correlation of up to twice the overlap (here ± 3 months or 90 days/7 days per week ≈ 13 weeks)^5^.

For the financial centres for which data are available, columns (1) to (4) of Table 2 summarise the results of the continental investment demand for London bills of exchange^6^. In particular, column (1) reports the OLS estimates of Eq. (3) for the Paris financial market. The coefficient estimate of 1.01 for the slope ($\widehat {\beta }$), which does not differ from a value of one at any conventionally used level of rejection, concurs almost perfectly with the prior of the interest-parity condition. This finding lends support to the abovementioned view that, around 1900, a highly active short-term investment demand for London bills of exchange aligned the discount and exchange rates between sterling and other core countries applying the gold standard. For the case of the Amsterdam financial market, the results in column (2) also lend empirical support to the interest-parity condition involving long-bills, in the sense of giving rise to an estimated slope coefficient $\widehat {\beta }$ that does not differ from one at conventional levels of statistical significance. Column (3) reports the results for Berlin. With a value of 0.96, $\widehat {\beta }$ is very close but, at the 5% level of statistical significance, different from one. Perhaps, this small discrepancy reflects that Berlin only began to emerge as important foreign exchange centre during the 1890s (compare “Weekly discount and exchange rate data” section)^7^. Similarly, for the investment demand for London bills of exchange from Brussels, the slope coefficient of 1.05 is close, but statistically different, from the hypothesised value. Recall from Fig. 2 that before 1902 sight-rates of the Belgian franc are often missing, wherefore only around 900 weekly observations are available^8^.

Transaction costs can drive a wedge into interest parity conditions (see, e.g. Engel 2014, p. 455). For the current example looking at the investment demand for bills of exchange, an ad-valorem stamp duty of $\frac {1}{2}$ per mille was typically levied on long-bills issued in London (see, e.g. Clare 1895, ch. 12). Depending on the country, the type of bill, or the value of a transaction, sight-bills were typically subject to smaller, or were even exempted from, stamp duties (see, e.g. Tate 1908). However, transaction costs of similar magnitude than the stamp duty could arguably arise from brokerage fees and insurance coverage (see Thomas 1921, pp. 194–195). Although the transaction costs differed between European countries (Thomas 1921, p. 24), they were typically smaller than those reported in Coleman (2012) for the New York financial market. Still, stamp duties and fees could be responsible for at least a part of the small deviations from the interest-parity condition found in columns (1) to (4) of Table 2.

To uncover whether the interest-parity condition held for the London investment demand for long-bills of exchange, the standard assumption that expectations correspond with future exchange rates, or $E\left [s_{t+m}^{*}\right ]=s_{t+m}^{*}$, is introduced (see Coleman 2012, p. 27). The maturity equals 3 months, or *m*=13 weeks. Hence, transforming (2) using logarithms yields

4 $$ l_{t}-s_{t+m}^{*}=\alpha + \beta(i_{t}) + \epsilon_{t}.  $$

Recall from the discussion above that (4) differs from (3) in terms of encapsulating an exchange-rate risk. However, the null hypothesis that the interest-parity condition as regards the London investment demand for bills of exchange holds requires that *β*=1.

Across the main financial centres on the European continent, columns (5) to (8) of Table 2 summarise the results involving a combined investment in long-bills issued in London with a sight-bill transaction on London upon maturity. With estimates between 0.62 and 0.81, the slope coefficients are much closer to the hypothesised value than commonly found with modern data when future exchange rate movements are left uncovered (see, e.g. Engel 2014, p. 495). Still, the hypothesis that *β*=1 is clearly rejected. This finding could reflect that before 1914, British banks were reluctant to invest in foreign currency bills. Unfortunately, the contemporaneous books give only vague reasons why this was the case, e.g. by merely mentioning that “foreign bills as an investment are strangely neglected by English bankers” (Clare 1902, p. 95). In any case, a key difference was that London investments in continental bills, according to (4), were subject to exchange-rate risk, whereas continental banks could cover this by a suitable long-bill transaction (see (3)).

During the classical gold standard, aggravated levels of political or economic uncertainty manifested themselves in large deviations of the sight-rate from the mint-par (Clare 1902, p. 98). To uncover how the investment demand for bills of exchange reacted to such events, Table 3 splits the sample into observations within and outside (or on) the gold points. Of note, the vagaries to determine the gold points introduce an important caveat to this approach. Still, for observations laying outside the gold points according to the values reported in the “Exchange rates during the gold standard” section, the top panel suggests that the interest-parity condition as regards the London investment demand for continental bills of exchange can no longer be rejected for the case of Paris (whereas there is only a rejection at the 10% level for the case of Amsterdam)^9^. Although the London banker was, reportedly, “content with a safe and steady rate of interest” (Thomas 1921, p. 78), sufficiently large opportunities to make a chance profit did, perhaps, give rise to more active short-term investment activity in higher yielding bills of exchange on established financial centres such as Paris. Conversely, according to the bottom panel, the results of (3) differ hardly when distinguishing between observations within and outside the gold points. For the continental investment demand for London bills of exchange, where exchange rate risk was hedged, this distinction should indeed be less relevant.

Taken together, in how far the interest-parity condition can be rejected for transactions between the major continental financial centres and London around 1900 seems to depend on such things as the deviation from the mint-par. In any case, it should not be overlooked that an interest-parity puzzle, in terms of massive deviations from the theoretically expected slope coefficient or even negative values for *β* that have frequently been found with modern data (see, e.g. Engel 2014, p. 495), does not arise in Tables 2 and 3. Perhaps thanks to the certainties associated with sufficiently stable exchange rates, when properly specified in terms of bills of exchange transactions, the interest-parity condition held remarkably well between the core currencies of the classical gold standard.

## Conclusion

This paper has examined the interest-parity condition during the “belle epoque” of the gold standard (1880–1914). The main innovation is to highlight the role of bills of exchange as financial instruments to carry out interest-parity transactions between sterling and four European currencies (French and Belgian franc, Dutch guilder, German mark) at the time. There are two main take-home messages.

Firstly, as early as the end of the nineteenth century, the premise that interest and exchange rates are closely intertwined was prominently discussed in a number of books about the foreign exchanges and money markets. Above all, among practitioners, it seems to have been widely known that European banks exploit international differences in the short-term return between the major gold-backed currencies. Yet, since international transactions occurred mainly by means of bills of exchange, the links between the exchange and interest rates in those days are, perhaps, no longer obvious to the modern eye.

Secondly, for the most advanced European financial centres around 1900, regressing the short-term interest from discounting bills in the local money markets onto the return implied in foreign bills of exchange yields coefficients that come at least close to the interest-parity condition. This lends support to the historical claim that the so-called investment demand for bills of exchange aligned the exchange rates of the major gold-backed currencies with the money market discount rates. Given that massive deviations from the interest-parity condition (in particular with the uncovered version) have commonly been found with modern data, this is a remarkable result.

## Footnotes

1 Rather than quantifying the numerous cost components of transporting gold across borders, the band of gold-arbitrage inactivity has also been estimated from the nonlinear time series behaviour of the exchange rate around the gold-points. For example, Canjels et al. (2004); Juhl et al. (2006), and Bernholz and Kugler (2011) employ a threshold autoregression model for this task.

2 Cheques are a specific type of a bill in terms of being a written order to a *bank* to pay a specified amount upon presentation (Cassis 2010, p. 296).

3 Given that New York began to emerge as an important financial centre (Goodhart 1969; Cassis 2010, pp. 114ff.), it is perhaps surprising that the United States are not mentioned as important participant in the London discount market. However, although the United States were a major trading partner for Britain, the capital flows were nowhere near those towards and from the principal European countries. The transaction costs of shifting financial capital across the Atlantic were still comparatively high. Hence, “the relative value of money, which is so largely responsible for the movements of the chief European exchanges, produces scarcely any perceptible effect on the American rate” (Clare 1902, p. 130).

4 During the second half of the 19th century, an early forward market had developed in Vienna to deal with the unstable behaviour of the Austrian-Hungarian currency, which was effectively on a flexible exchange rate until the 1890s (Flandreau and Komlos 2006). It is therefore, perhaps, not surprising that for the case of Austria-Hungary, a verbal analysis of covered interest-arbitrage transactions between Vienna and Berlin can be found as early as Lotz (1889, pp. 1277ff.). Furthermore, around 1900, another active forward exchange market existed for the Russian rouble (see Yeager 1969).

5 A Bartlett-kernel is used and there is no pre-whitening of the residuals. Note that changing these options, or even altering the bandwidth to values slightly different from 13, did not overturn the essence of the results.

6 For all variables of Table 2, the conventional unit-root tests (ADF, Phillips-Perron) suggest that even at the 1 per cent level of rejection, the time series are stationary (for the sake of brevity, the corresponding results are not reported here, but are available on request).

**Table 2:** Results: Interest parity via long-bills of exchange

	Continental investment demand for London bills of exchange					London investment demand for continental bills of exchange			
	Paris	Amsterdam	Berlin	Brussels		London on			
	on London					Paris	Amsterdam	Berlin	Brussels
	(1)	(2)	(3)	(4)		(5)	(6)	(7)	(8)
Intercept $\left (\widehat {\alpha }\right)$	0.003	0.01	0.01	0.001		0.01	0.01	0.01	0.01
	(0.0004)	(0.001)	(0.001)	(0.001)		(0.002)	(0.002)	(0.002)	(0.003)
*i* $_{t}^{*} \left (\widehat {\beta }\right)$	1.01	0.97	0.96	1.05					
	(0.02)	(0.02)	(0.02)	(0.02)					
*i* $_{t} \left (\widehat {\beta }\right)$						0.62	0.75	0.81	0.75
						(0.05)	(0.07)	(0.06)	(0.09)
Reject (*β*=1)			**	***		***	***	***	***
*R* ^2^	0.81	0.84	0.87	0.90		0.30	0.33	0.39	0.37
*N*	1772	1759	1771	903		1759	1749	1759	890

Notes: This table reports estimates of Eq. (3) with dependent variable $l_{t}-s_{t}^{*}$ and (4) with dependent variable $l_{t}-s_{t+m}^{*}$ (expressed as annualised value). Estimation is by OLS with heteroscedasticity and autocorrelation robust (Newey-West) standard errors with a fixed bandwidth of 13 leads and lags. The null hypothesis that the interest parity (via long-bill transactions) holds implies that *β*=1*N* the number of observations Significant deviations from this are indicated by ** at the 5% level, and *** at the 1% level

7 Concurring with this, for the case of Berlin, the hypothesis that *β*=1 can no longer be rejected when restricting the sample to observations after 1900.

8 In principle, the Vienna financial market would also provide some data to test the interest parity via long-bills of exchange transactions. However, the monetary reforms mentioned back in the “Exchange rates during the gold standard” section give rise to structural breaks in the exchange rate data. Furthermore, Austria-Hungary only managed to gradually stabilise its exchange rate to gold, and was neither officially on the gold standard—banknotes remained inconvertible (see Von Mises 1909)—nor considered to be an active participant in the investment demand for bills of exchange (see Clare 1902, p. 94). It is therefore not surprising that, between London and Vienna, the interest-parity condition holds only when restricting the sample to some parts of the early 20th century. For all these reasons, the case of Austria-Hungary is not pursued further.

9 With deviations between 0.2 and 0.7 per cent from the mint-par, the bandwidth of gold-arbitrage inactivity with the gold-points reported in the “Exchange rates during the gold standard” section is similar to e.g. the 0.25 per cent reported for the sterling-to-guilder exchange rate around 1900 in Kugler (2016). The value of the gold-points can also be inferred from the nonlinear behaviour of the sight-rate $S_{t}^{*}$ (see footnote 1). Employing the threshold autoregression model of Canjels et al. (2004, p. 876) yields indeed slightly different gold-points of 25.10 Fcs/£ and 25.34 Fcs/£ for the French franc, 25.15 Fcs/£ and 25.29 Fcs/£ for the Belgian franc, 12.03 Fl/£ and 12.11 Fl/£ for the Dutch guilder, and 20.34 M/£ and 20.52 M./£ for the German mark. However, using these values did not change the essence of the results reported in Table 3.

**Table 3:** The effect of the gold-points

	Paris	Amsterdam	Berlin	Brussels	Paris	Amsterdam	Berlin	Brussels
Sample	Observations inside gold points				Observations outside gold points			
	(1)	(2)	(3)	(4)	(5)	(6)	(7)	(8)
London investment demand for continental bills of exchange								
Intercept ($\widehat {\alpha }$)	0.01	0.01	0.02	0.01	0.01	0.01	−0.002	0.02
	(0.002)	(0.002)	(0.002)	(0.004)	(0.01)	(0.005)	(0.01)	(0.01)
*i*_*t*_ ($\widehat {\beta }$)	0.58	0.73	0.77	0.75	0.81	0.69	1.34	0.53
	(0.05)	(0.07)	(0.06)	(0.12)	(0.17)	(0.16)	(0.11)	(0.16)
Reject (*β*=1)	***	***	***	**		*	***	***
*R* ^2^	0.32	0.33	0.36	0.39	0.24	0.23	0.80	0.18
*N*	1510	1559	1715	582	249	177	43	263
Continental investment demand for London bills of exchange								
Intercept ($\widehat {\alpha }$)	0.003	0.01	0.01	0.0002	0.004	0.01	0.02	0.001
	(0.0003)	(0.001)	(0.001)	(0.001)	(0.001)	(0.002)	(0.004)	(0.002)
*i*$_{t}^{*}$ ($\widehat {\beta }$)	1.02	0.98	0.96	1.07	0.97	0.95	0.71	1.03
	(0.02)	(0.02)	(0.02)	(0.02)	(0.06)	(0.09)	(0.09)	(0.05)
Reject (*β*=1)			**	***			***	
*R* ^2^	0.87	0.87	0.88	0.90	0.48	0.62	0.73	0.87
*N*	1520	1581	1728	615	252	178	43	288

Notes: The top and bottom panel of this table report estimates of, respectively, Eq. (4) with dependent variable $l_{t}-s_{t+m}^{*}$ and Eq. (3) with dependent variable $l_{t}- s_{t}^{*}$ (expressed as annualised value). The maturity *m* equals 3-months, that is 90 days or 90 days/7 days per week ≈ 13 weeks. Estimation is by OLS with heteroscedasticity and autocorrelation robust (Newey-West) standard errors with a fixed bandwidth of 13 leads and lags. According to Section “Exchange rates during the gold standard”, the gold-points of $S_{t}^{*}$ are 25.12 Fcs./ *$* and 25.32 Fcs./ *$* for France and Belgium, 12.05 Fl./ *$* and 12.15 Fl./ *$* for the Netherlands, as well as 20.32 M/ *$* and 20.53 M/ *$* for Germany. The null hypothesis that the interest parity (via long-bill transactions) holds implies that *β*=1. *N* denotes the number of observations. Significant deviations from this are indicated by * at the 10% level; ** at the 5% level, and *** at the 1% level
